# HMGA proteins as modulators of chromatin structure during transcriptional activation

**DOI:** 10.3389/fcell.2014.00005

**Published:** 2014-03-06

**Authors:** Nihan Ozturk, Indrabahadur Singh, Aditi Mehta, Thomas Braun, Guillermo Barreto

**Affiliations:** ^1^LOEWE Research Group Lung Cancer Epigenetic, Max-Planck-Institute for Heart and Lung ResearchBad Nauheim, Germany; ^2^Department of Cardiac Development and Remodeling, Max-Planck-Institute for Heart and Lung ResearchBad Nauheim, Germany

**Keywords:** HMGA, chromatin structure, transcription, development and cancer

## Abstract

High mobility group (HMG) proteins are the most abundant non-histone chromatin associated proteins. HMG proteins bind to DNA and nucleosome and alter the structure of chromatin locally and globally. Accessibility to DNA within chromatin is a central factor that affects DNA-dependent nuclear processes, such as transcription, replication, recombination, and repair. HMG proteins associate with different multi-protein complexes to regulate these processes by mediating accessibility to DNA. HMG proteins can be subdivided into three families: HMGA, HMGB, and HMGN. In this review, we will focus on recent advances in understanding the function of HMGA family members, specifically their role in gene transcription regulation during development and cancer.

## Introduction

**Figure 1:**
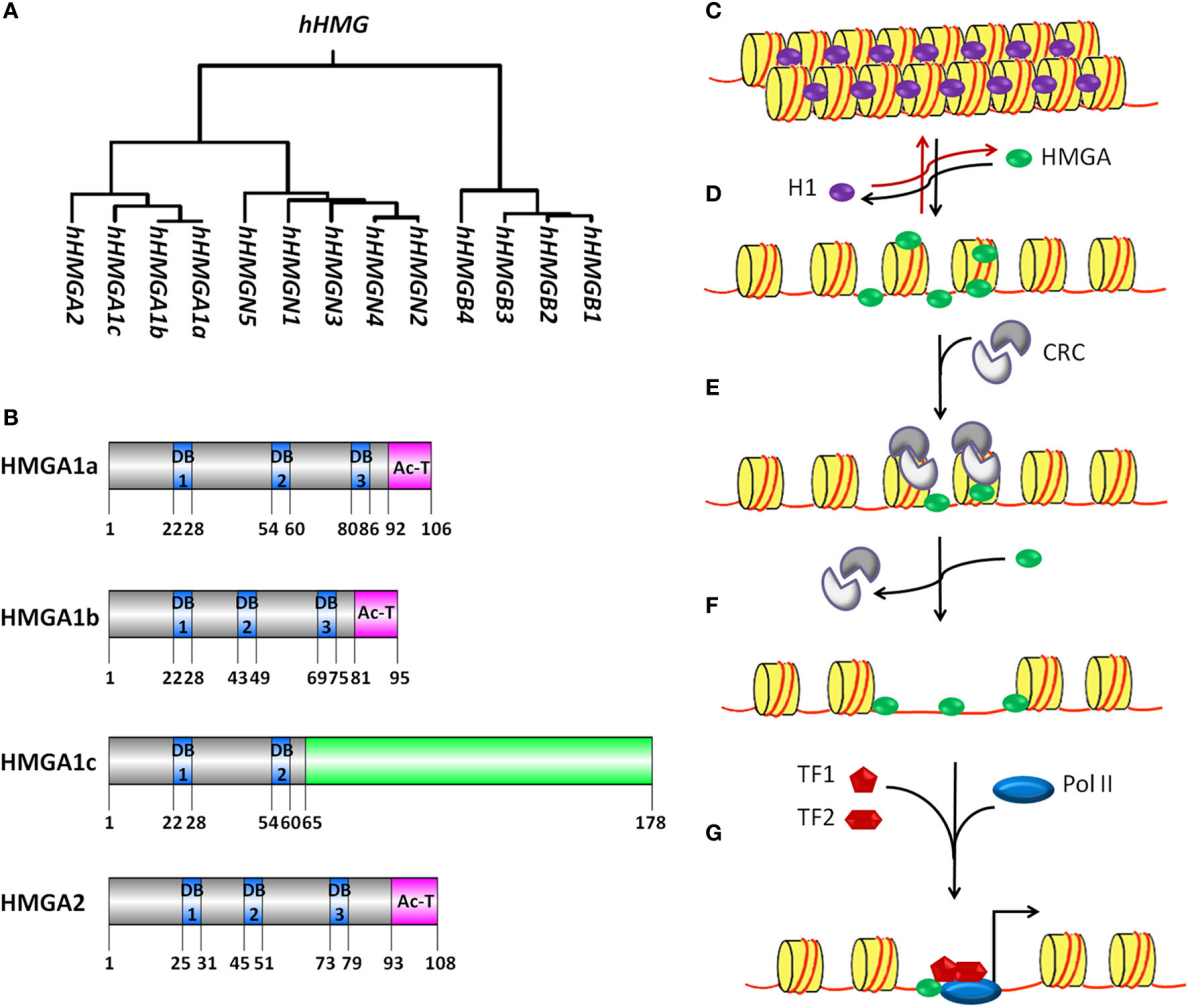
**The HMG proteins superfamily and model for HMGA proteins mediated transcription activation. (A)** Genealogical tree of human HMG proteins. HMG proteins are divided into three families depending on their DNA binding domains: HMGA (containing AT-hooks), HMGB (containing HMG-boxes) and HMGN (containing nucleosomal binding domains). CLUSTALW software was used to build rooted genealogical tree with branch length (UPGMA) of human HMG group family members. **(B)** Schematic representation of human HMGA proteins. HMGA1a, HMGA1b, HMGA1c, and HMGA2 proteins contain unique DNA binding domains (DB, blue boxes) with a characteristic AT-hook motif and a C terminal acidic domain (pink boxes). HMGA1a and HMGA1b proteins show high sequence similarity. HMGA1b lacks eleven amino acids before the second DB when compared to HMGA1a and 1c. The amino acid sequence of HMGA1c differs from the one from HGMA1a and 1b starting from amino acid 65 (green box). GPS DOG 2.0 software was used to illustrate protein structure domains (Ren et al., 2009). **(C–G)** A model for the putative mechanism of HMGA proteins as factors promoting nucleosome mobility and accessibility to specific DNA sites for transcription activation is depicted. **(C)** The nucleosome is built of 146 bp DNA (red line) surrounding the histone octamer (yellow cylinder), which consist of two H2A-H2B dimers and one (H3–H4)_2_ tetramer. The linker histone H1 (purple oval) binds to linker DNA outside the histone octamer at the position where the DNA enters and exits the nucleosome core particle. **(D)** HMGA proteins (green oval) can compete and displace histone H1 from the chromatin. HMGA binding to linker DNA in the chromatin and the subsequent displacement of histone H1 leads to decompactness of the chromatin. **(E)** The relaxed chromatin structure acts as an anchoring site for the recruitment of chromatin remodeling complexes (CRC, gray). Binding of this complex is enhanced by HMGA proteins and induces eviction of core histones and/or mobilization of complete histone octamers. **(F)** The chromatin remodeling events make sequence-specific sites on the DNA accessible for transcription factor (TF, red pentagon and hexagon) binding. **(G)** HMGA proteins might facilitate the formation of transcription factor complexes binding to these sequence-specific sites. Later, RNA polymerase II (Pol II, blue oval) is recruited for transcription initiation (black arrow).

Chromatin constitutes the physiological template for transcription, thereby increasing the complexity of gene transcription regulation. Nucleosomes are the structural and functional units of chromatin. A nucleosome is built of DNA surrounding a histone octamer, which consists of two H2A–H2B dimers and one (H3–H4)_2_ tetramer. In addition to nucleosomes, chromatin consists of non-histone chromatin-associated proteins. It is well known that chromatin mediated regulation of transcription involves DNA methylation and histone modifications (Zhang et al., 2005; Karlic et al., 2010; Ha et al., 2011; Gohlke et al., 2013). However the precise biological function of non-histone chromatin-associated proteins is not yet clear. High mobility group (HMG) proteins are the most abundant non-histone chromatin associated proteins. Based on their DNA binding domains, the HMG proteins are subdivided into three families (Figure 1A): HMGA (containing AT-hooks), HMGB (containing HMG-boxes) and HMGN (containing nucleosomal binding domains) (Bustin, 2001; Catez and Hock, 2010). HMG proteins can recognize structure rather than a particular nucleotide sequence and are able to bind to specific structures in DNA or chromatin in a sequence-independent fashion via their respective functional motifs (Reeves, 2001). HMG proteins do not possess intrinsic transcriptional activity while having the ability to modulate transcription of their target genes by altering the chromatin structure at the promoter and/or enhancers (Reeves, 2010). Thus, they are called architectural transcription factors. In this review, we will focus on recent advances in understanding the function of HMGA family members. HMGA family is comprised of HMGA1a, HMGA1b, HMGA1c, and HMGA2. They are expressed highly during embryonic development and in transformed cells. They are encoded by two distinct genes. The *HMGA1* gene gives rise to three proteins (HMGA1a, HMGA1b, and HMGA1c) by alternative splicing of a common transcript. With the exception of HMGA1c, the HMGA proteins contain three short basic repeats called AT-hook motif and a C-terminal acidic tail (Figure 1B) (Sgarra et al., 2004; Fusco and Fedele, 2007; Hammond and Sharpless, 2008; Pfannkuche et al., 2009). The amino acid sequence of the AT-hook motif is K/RXRGRP (X = glycine or proline) and is flanked on either side by positively charged residues. These characteristics determine the binding preference of HMGA proteins to both the minor groove of AT-rich DNA stretches (Cui and Leng, 2007; Winter et al., 2011) and to nucleosomes in a cooperative manner (Li et al., 2006). HMGA proteins, when free in solution, possess very little, if any, secondary structure as shown by biophysical techniques such as circular dichroism (CD) (Lehn et al., 1988) and nuclear magnetic resonance (NMR) spectroscopy (Evans et al., 1995). However, when bound to DNA or proteins specifically, the AT-hooks undergo from a disordered to ordered conformational change and influence the conformation of bound DNA substrates in different ways including bending, straightening, unwinding, and inducing looping in linear DNA molecules. The function of the C-terminal acidic region is poorly understood. However, there are speculations that C-terminal acidic tail is involved in protein-protein interaction and recruitment of factors during regulation of gene transcription (Xu et al., 2011).

HMGA family members show high sequence homology; the only difference between HMGA1a and HMGA1b is an internal deletion of eleven amino acids before the second AT-hook domain. Although the sequence similarity may suggest that these two isoforms could be biologically interchangeable, there are reports that show the opposite. For example, it was shown that tetracycline-regulated induction of *HMGA1b* in the human breast epithelial cell line MCF7 caused them to progress much more rapidly to a metastatic and highly malignant phenotype than did induced overexpression of *HMGA1a* (Reeves et al., 2001). The differences in their posttranslational modifications may explain the different cellular processes they are involved in. Also the different spacing of AT-hook domains between the different HMGA proteins could have a role in their different target gene selection and/or binding affinity (Cleynen and Van De Ven, 2008).

## HMGA and chromatin

In the nucleus of eukaryotic cells, genomic DNA is highly organized and packed into chromatin. Chromatin serves as substrate for all kinds of DNA dependent processes and represents a strong barrier to sequence specific recognition sites on the DNA. Thus, to overcome this DNA sequence accessibility barrier, it is a prerequisite to open the higher order chromatin structure so that transcription regulators can access to specific DNA sequences and execute their function. Histone H1 binds to linker DNA and increases the compactness of higher order chromatin (Figures 1C,D). HMGA proteins compete with histone H1 for binding to linker DNA thereby inducing a loosening of the chromatin structure, which has been demonstrated by different techniques including fluorescence recovery after photobleaching (FRAP), nuclear fractionation analysis and micrococcal nuclease digestion (MNase) assays (Zhao et al., 1993; Catez et al., 2004; Kishi et al., 2012). However, the molecular mechanism underlying the replacement of histone H1 by HMGA proteins which results in chromatin opening is not well understood. Several post-translational modifications have been reported for histone H1, such as phosphorylation, methylation, acetylation and poly-ADP-ribosylation (PARylation) (Snijders et al., 2008). Similar to the core histones, these post-translational modifications of the linker histone H1 play a role in regulation of chromatin structure. PARP-1 mediated PARylation of histone H1 leads to nucleosome-specific exchange of histone H1 by HMGB proteins inducing local changes of chromatin structure (Ju et al., 2006). In a similar manner, histone H1 post-translational modifications could facilitate replacement of histone H1 by HMGA proteins thereby inducing chromatin decompaction. It is well known that the globular domain of histone H1 interacts and binds with linker DNA. Several motifs for kinases are located inside the globular domain or flanking it. Phosphorylation of these sites modulate the binding affinity of histone H1 to linker DNA (Contreras et al., 2003) and might play a role during replacement of histone H1 by HMGA proteins.

Histone H1 eviction from the chromatin is not enough to facilitate the access of regulatory elements on target genes because the DNA is still wrapped around the core histones hindering the accessibility of transcription factors to their binding elements. Hence, there is further need of either eviction or mobilization of core histones (Figures 1E,F). Several chromatin remodelers might be involved in these processes. For example, the FACT complex has been reported to participate in H2A/H2B histone eviction/deposition (Belotserkovskaya et al., 2003), whereas ASF1 is involved in H3/H4 histone eviction/deposition (Schwabish and Struhl, 2006). HMGA proteins bind to both nucleosomes (Li et al., 2006) and chromatin remodelers (Malini et al., 2011) suggesting a role of these proteins in eviction and/or mobilization of core histones during transcriptional regulation.

## HMGA and transcription

In eukaryotes, two types of core promoters are used for initiation of gene transcription: promoters that are enriched with the di-nucleotide sequence CpG and promoters that are CpG poor (Antequera, 2003; Ramirez-Carrozzi et al., 2009; Deaton and Bird, 2011). CpG poor core promoters usually contain a TATA box, initiator sequences (INR), a TFIIB recognition element (BRE) and downstream promoter elements (DPE) (Butler and Kadonaga, 2002; Levine and Tjian, 2003). These promoters have specific single transcription start sites (TSS). In addition, a strong stimulation of RNA polymerase II (Pol II) dependent transcription initiation has been reported for these promoters by the synergistic interplay of the TATA box and INR core promoter elements. Recently, HMGA1 was identified as one of the factors required for the synergy between the TATA box and INR elements (Figure 1G) (Xu et al., 2011). However, there is still need of further investigation to test the relevance of this finding as a general mechanism of transcription initiation. Gene transcription can also be regulated by the interplay of core promoter elements along with regulatory DNA elements, such as enhancers and silencers, which might be located several kilo base pairs (kbp) upstream or downstream of the promoter. In response to defined signals, specific proteins bind to the enhancer and form a complex called enhanceosome (Munshi et al., 1999; Yie et al., 1999; Panne et al., 2007; Panne, 2008). Looping of the DNA brings the enhaceosome and the core promoter in close proximity resulting in enhanced gene transcription. HMGA proteins are involved in enhanceosome formation (Bouallaga et al., 2000, 2003). In addition, it has been reported that HMGA proteins have the ability to bend DNA (Chen et al., 2010). Thus, a model can be suggested in which HMGA proteins participate not only in the formation of the enhanceosome but also in DNA looping and chromatin rearrangements that occur to bring enhanceosomes and core promoter in close proximity so that a coordinated assembly of the transcription initiation complex at core promoter can take place.

It has been reported that the C-terminal domain of HMGA proteins (Figure 1B) is required for the interaction of HMGA1 with TFIID (Xu et al., 2011). This interaction seems to mediate core promoter specific functions since a C-terminal deletion mutant of HMGA1 fails to initiate transcription (Figure 1G). In addition, this domain contains several conserved phosphorylation sites, for example SQ sites, which are substrates for the kinases DNA-PK, ATM and ATR. These kinases have been involved in different signal transduction pathways. In addition, ATM and ATR interact with and phosphorylate HMGA group proteins (Pentimalli et al., 2008; Palmieri et al., 2011; Natarajan et al., 2013). It would be interesting to elucidate the biological relevance of this finding within the context of different signal transduction pathways to understand the signaling events responsible for fine-tuning the HMGA mediated cell specific gene transcription.

The second type of promoters, which contain CpG islands, lack TATA boxes and display multiple heterogeneous TSSs. These promoters tend to be enriched with binding elements for SP1, NRF-1, E2F, and ETS transcription factors (Landolin et al., 2010; Deaton and Bird, 2011). It was shown that HMGA1 interacts with SP1 and facilitates its binding to both the human insulin receptor (*INSR*) gene promoter and the herpes simplex virus latency-active promoter 2 (Figure 1G) (French et al., 1996; Foti et al., 2003). This suggests that HMGA family members may be involved in the transcriptional activation of CpG rich promoters. Although previous reports support the specific binding of HMGA to AT-rich sequences on the DNA (Cleynen et al., 2007; Cui and Leng, 2007; Winter et al., 2011), it would be interesting to compare transcriptome data after HMGA gain-of-function with whole-genome chromatin immunoprecipitation sequencing (ChIP-seq) data after HMGA-ChIP to find out whether HMGA mediated regulation of CpG rich promoter is a general mechanism or it is only restricted to specific genes.

## HMGA role during development

**Figure 2:**
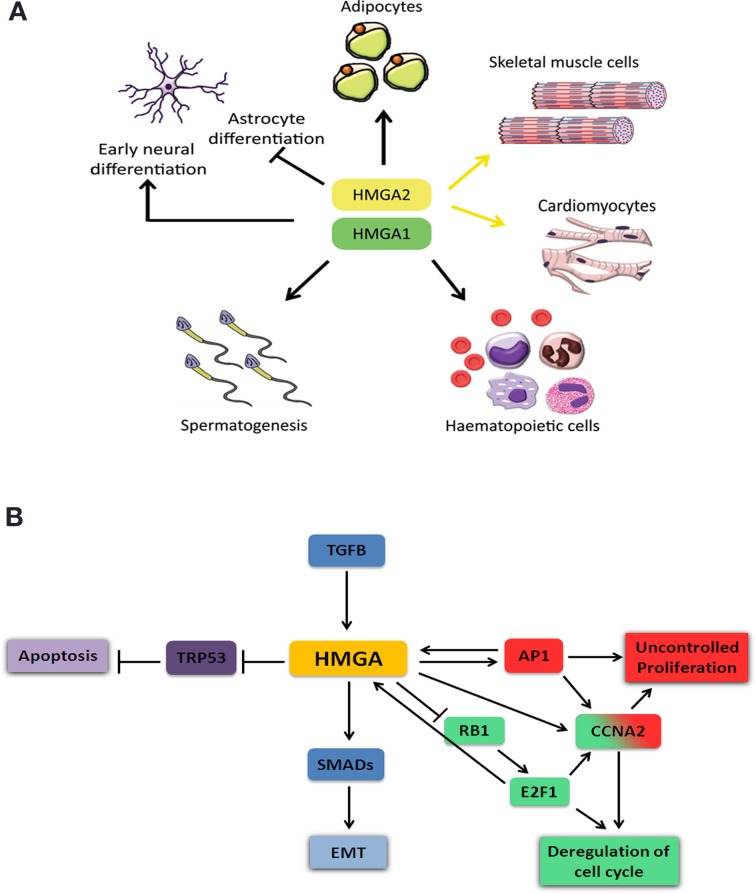
**Schematic representation of HMGA role during cell differentiation and cancer. (A)** HMGA proteins are required for proper development and progenitor cell differentiation to adipocyte, skeletal muscle, cardiac muscle, spermatozoids, and hematopoietic cells. They have a dual role in neuronal differentiation, where they are required during early neural progenitor differentiation but inhibit astrocyte differentiation at later stages. **(B)** HMGA proteins are involved in cellular processes that induce tumor formation and metastasis. Abnormal high expression of HMGA proteins results in tumor formation and metastasis. HMGA proteins deregulate cell cycle and thereby increase cell proliferation through either inhibition of RB1 and induction of E2F1 (green) or activation of AP1 and increased Cyclin A2 (*Ccna2*) expression (red). HMGA interaction with TRP53 blocks its activity and results in inhibition of apoptosis (purple). TGFB positively regulates *HMGA* expression (blue). HMGA interaction with SMADs leads to epithelial mesenchymal transition (EMT).

HMGA proteins are present at high levels in various undifferentiated tissues during embryonic development and their levels are strongly reduced, or almost absent in case of HMGA2, in the corresponding adult tissues (Pfannkuche et al., 2009). *Hmga1* and *Hmga2* are highly and ubiquitously expressed during early embryonic development (Chiappetta et al., 1996; Hirning-Folz et al., 1998). At later stages, *Hmga1* expression becomes restricted to specific organs of all three germ layers, the ectoderm, mesoderm and endoderm. Although *Hmga1* can be detected in most adult tissues, its expression is higher in testis, skeletal muscle, and thymus (Chiappetta et al., 1996). *Hmga2* expression is mainly limited to the mesenchyme and is hardly detectable in healthy adult tissues, except testis, skeletal muscle and adipose tissues (Anand and Chada, 2000; Chieffi et al., 2002; Caron et al., 2005). HMGA proteins in adult organs have been implicated in maintaining and activating stem/progenitor cells in different tissues (Figure 2A) (Nishino et al., 2008; Li et al., 2012). When compared to wild-type embryonic stem (ES) cells, *Hmga1*^−/−^ ES cells form fewer and smaller embryoid bodies (EBs) and show reduced capacity to differentiate to T lymphocytes (Caron et al., 2005). *Hmga1*^−/−^ mice suffer from hematologic malignancies and cardiac hypertrophy resulting from a direct regulation of cardiomyocyte growth (Figure 2A) (Fedele et al., 2006a). In addition, *Hmga1*^−/−^ mice show reduced insulin receptor expression leading to type 2 diabetes (Foti et al., 2005; Fedele et al., 2006a). The interaction of HMGA1 with retinoblastoma protein (RB1, also known as pRB) and CCAAT/enhancer-binding protein β (C/EBPβ) was shown to be important for proper regulation of genes required for adipocyte differentiation (Figure 2A) (Esposito et al., 2009). Transgenic mice carrying an *Hmga1b* truncated gene exhibit gigantism with an increased amount of the retroperitoneal and subcutaneous white adipose tissue, possibly due to activation of E2F transcription factor 1 (E2F1) pathway. These mice also develop B-cell lymphomas similar to that occurring in *Hmga1*^−/−^ mice (Fedele et al., 2011).

*Hmga2*^−/−^ mice show a pygmy phenotype due to reduced expression of the insulin-like growth factor 2 mRNA binding protein 2 (*Igf2bp2*) gene (Zhou et al., 1995; Brants et al., 2004; Cleynen et al., 2007; Li et al., 2012). The *Hmga2* deficient mice also show reduced fat tissue which is linked to a function of *Hmga2* in pre-adipocyte precursor cell proliferation (Figure 2A) (Anand and Chada, 2000). In addition, *Hmga2*^−/−^ embryonic fibroblasts have a proliferative defect. A recent study shows that *Hmga2* is extremely important for the self-renewal potential of hematopoietic stem cells (HSCs, Figure 2A) (Copley et al., 2013). On the other hand, transgenic mice overexpressing a carboxyl-terminally truncated version of *Hmga2* show a giant phenotype, are obese and develop lymphomas (Baldassarre et al., 2001; Fedele et al., 2001). It was shown that HMGA2 is necessary for the commitment of mouse embryonic stem cells to the skeletal muscle (Figure 2A) (Caron et al., 2005; Li et al., 2012) and cardiac muscle lineages (Figure 2A) (Monzen et al., 2008). HMGA proteins are also important for the proliferation of early stage neural precursor cells (NPCs) and for their neurogenic potential and overexpression of these genes can reprogram late stage NPCs into cells with early stage-specific capacities suppressing astrogenesis (Figure 2A) (Kishi et al., 2012). HMGA proteins are required for normal sperm development; both *Hmga1*^−/−^ and *Hmga2*^−/−^ mice show impaired spermatogenesis and the latter are sterile (Figure 2A) (Chieffi et al., 2002; Liu et al., 2003).

## HMGA proteins and cancer

*Hmga1* and *Hmga2* are highly expressed in transformed cells in a variety of malignant and benign tumors of different origins (Fusco and Fedele, 2007; Hock et al., 2007). Transformation of a rat thyroid cell line (FRTL5) by the Kirsten murine sarcoma virus (KiMSV) increased sharply the level of phosphorylated HMGA proteins along with phosphorylated histone proteins H1 and H2A, and ubiquitinated H2A. Thus, the association of HMGA proteins with the neoplastic phenotype was demonstrated for the first time (Giancotti et al., 1985). Subsequently, a direct role of HMGA proteins in tumorigenesis was proven with a loss of function of HMGA2 in normal rat thyroid cells which prevented retrovirally induced neoplastic transformation (Berlingieri et al., 1995). More direct evidence of the tumorigenic potential of HMGA proteins came when normal cells in culture overexpressing these proteins underwent oncogenic transformation (Wood et al., 2000). For example, the human breast epithelial cell line MCF7 acquired the ability to form both primary and metastatic tumors in nude mice after overexpression of *HMGA1* (Reeves et al., 2001). In general, there are accumulating reports supporting that HMGA proteins are required and sufficient for neoplastic transformation (Di Cello et al., 2008; Winslow et al., 2011; Morishita et al., 2013; Sun et al., 2013).

HMGA proteins contribute to tumor formation by inhibiting the apoptotic function of the transformation related protein 53 (TRP53, also known as P53) (Figure 2B). Two mechanisms have been reported for this inhibition. HMGA1 directly binds to and inactivates TRP53 thereby modulating the transcription of its target genes *Cdkn1a* (cyclin-dependent kinase inhibitor 1A, also known as p21), *Bcl2* (B cell leukemia/lymphoma 2) and *Bax* (Bcl2-associated X protein and cyclin-dependent kinase inhibitor 1A), while enabling the active transcription of the TRP53 inhibitor *Mdm2* (Frasca et al., 2006; Pierantoni et al., 2006; Esposito et al., 2010, 2012). The second mechanism is indirect and involves translocation of HIPK2 (homeodomain-interacting protein kinase 2) to the cytoplasm. HIPK2 phosphorylates p53 at S46 in the nucleus to induce apoptosis after non-repairable DNA damage has taken place (Pierantoni et al., 2007). The cytoplasmic translocation of HIPK2 abolishes the apoptotic function of TRP53.

Another mechanism by which HMGA proteins exert their tumorigenic activity is by interfering with cell cycle (Figure 2B). RB1 is one of the main cell cycle regulator proteins and keeps the E2F1 inactive in a complex with HDAC1 resulting in transcriptional repression. HMGA2 is able to bind RB1 and displace HDAC1 from the complex. Subsequent recruitment of the histone acetyl transferase EP300 (E1A binding protein p300, also known as P300) to the complex results in acetylation of DNA associated histones. E2F1 leads to activation of the protein complex and target gene transcription (Fedele et al., 2006b). The activation of E2F1 mediated by HMGA2 was shown to induce pituitary adenomas. Interestingly, a recent study has shown that human *HMGA1* promoter is target of E2F1 and the deregulated RB1/E2F1 pathway might contribute to deregulation of HMGA1 in cancer (Figure 2B) (Massimi et al., 2013).

HMGA proteins interfere with cell cycle also by regulating the expression of Cyclin A2 (*Ccna2*) gene through the E2F1 (Schulze et al., 1995) and AP1 complexes (Casalino et al., 2007) (Figure 2B). AP1 complex can be composed of three Jun proteins (JUN, JUNB and JUND) and four Fos proteins (FOS, FOSB, FRA1, and FRA2). It was reported that HMGA protein levels increased in RAS-transformed rat thyroid cells, which in return increased the transcription of *Junb* and *Fra1* (Vallone et al., 1997). In these transformed cells, AP1 complex is mainly composed of JUNB-FRA1 heterodimers, and this compositional change leads to increased transcriptional activity of the AP1 complex explaining the high levels of *Ccna2*.

During epithelial-mesenchymal transition (EMT), polarized epithelial cells undergo several biochemical processes that result in a transition to a mesenchymal cell phenotype (Thiery and Sleeman, 2006; Kalluri and Weinberg, 2009). These processes include altered cell-cell and cell-extracellular matrix (ECM) interactions and reorganization of the cytoskeleton, both leading to an increased ability of the cells to migrate. Although EMT occurs during normal embryonic development, it is also a key process during tumorigenesis and metastasis. The transforming growth factor beta (TGFB) signaling pathway is able to induce EMT and has been involved in cancer initiation and metastasis. TGFB increases the expression of *Hmga2* by SMAD3 mediated inhibition of *let-7*, a microRNA that targets *Hmga2* transcript (Lee and Dutta, 2007). HMGA2 was found to be required for TGFB signaling and to interact with several SMAD proteins which are mediators of the TGFB signaling (Figure 2B) (Thuault et al., 2006, 2008; Watanabe et al., 2009; Wu et al., 2011; Zha et al., 2012). Moreover, HMGA and SMADs have been shown to coregulate the expression of *SNAIL1* (Protein snail homolog 1), which is the master effector for the induction of EMT in mammary epithelial cells (Thuault et al., 2008). Taking all these together, it will be of interest from the therapeutic point of view to investigate whether HMGA proteins are required for TGFB induced EMT during tumorigenesis and metastasis. Indeed, the multiple roles of TGFB signaling in tumor progression have promoted the development of therapeutic agents based on the inhibition of this signaling pathway (Golestaneh and Mishra, 2005; Ikushima et al., 2009). Interestingly, it has been recently shown that HMGA2 increased the expression of the TGFB type II receptor thereby enhancing TGFB signaling in epithelial carcinomas (Morishita et al., 2013).

LIN-28, a developmentally regulated RNA binding protein which is expressed at high levels in undifferentiated ES cells, has been shown to inhibit *let-7* microRNA biogenesis (Thornton and Gregory, 2012).The balance between LIN-28 and *let-7* affects the expression of *Hmga2* and therefore is important for ES cell self-renewal and differentiation (Li et al., 2012; Copley et al., 2013) and maintenance of an undifferentiated state in cancer cells (Shell et al., 2007). Moreover, a recent study has shown that *Hmga2* could promote cancer progression by acting as a competing endogeneous RNA (ceRNA) for *let-7*, adding another complexity to the mechanism by which *Hmga2* can contribute to tumorigenesis (Kumar et al., 2013). LIN-28/*let-7* axis also regulates glucose uptake (Zhu et al., 2011) and has the ability to reprogram cells toward glycolytic metabolism (Thornton and Gregory, 2012). This different metabolism, which is a hallmark of cancer, is known as the “Warburg Effect.”

Chromosomal rearrangements have also been reported in a variety of common benign tumors. Among these, rearrangements of the *HMGA2* gene at 12q15 seem to be the most common rearrangements in human tumors of mesenchymal origin. The role of *HMGA* translocations in the formation of various human tumors has been previously reviewed (Fusco and Fedele, 2007; Wu and Wei, 2013).

## Conclusions and perspective

High mobility group (HMG) proteins are the most abundant non-histone chromatin associated proteins. Members of the HMGA subfamily contain a unique DNA-binding domain, the AT-hook, which can bind AT-rich DNA sequences with high affinity. HMGA proteins act as architectural transcription factors, which means that although they do not possess intrinsic transcriptional activity, they have the ability to modulate transcription of their target genes by altering the chromatin structure in different ways: displacement of histone H1 leading to chromatin decompaction, high-order chromatin rearrangements resulting in DNA looping that brings enhancers and promoter in close proximity, promoting assembly of regulatory nucleoprotein complexes, such as enhanceosomes and transcription initiation complexes, facilitating nucleosome remodeling and thereby accessibility of DNA to transcription factors and recruiting transcription factors to specific promoters.

HMGA proteins undergo several post-translational modifications, such as phosphorylation (Nissen et al., 1991; Wang et al., 1995; Schwanbeck et al., 2000; Pierantoni et al., 2001; Di Agostino et al., 2004; Zhang and Wang, 2007), methylation (Sgarra et al., 2003; Edberg et al., 2004, 2005; Sgarra et al., 2006), acetylation (Munshi et al., 1998, 2001; Zhang et al., 2007), sumoylation (Cao et al., 2008) and ribosylation (Elton and Reeves, 1986). Here, we have discussed only the role of phosphorylation of HMGA proteins mediated by the kinases DNA-PK, ATM and ATR at the SQ sites during transcriptional regulation. The biological relevance of the other modifications is not well understood and requires more attention. It would be interesting to analyze also the other post-translational modifications to understand the molecular mechanisms underlying their role in fine-tuning the interaction of HMGA proteins with other transcription factors, with the chromatin and with the DNA.

HMGA proteins are present at high levels in various undifferentiated tissues and several studies confirmed their central role during normal embryonic development. They contribute to the plasticity of ES cells by maintaining an open chromatin state evicting linker histone H1 which facilitates formation of nucleoprotein structures at specific promoters (Pfannkuche et al., 2009; Kishi et al., 2012). Hence, it is not surprising that HMGA protein levels are developmentally regulated; they are strongly reduced or almost absent in adult organs, where they have been implicated in maintaining and activating stem/progenitor cells in different tissues. Regeneration of adult organs during normal homeostatic turnover or after injury requires a proper balance between self-renewal and differentiation of tissue-specific progenitor cells. On the other hand, re-expression of both *Hmga1* and *Hmga2* at high levels in the adult stage is seen in a variety of malignant and benign tumors, which might be in part due to their role in transcription activation of certain cell proliferation genes. Characterization of the role of HMGA proteins in the regulatory mechanisms controlling the proper balance between expansion and differentiation of progenitor cells will have a profound impact on our understanding and treatment of diseases originated from improper regulation of HMGA proteins, such as cancer, diabetes and idiopathic pulmonary fibrosis (Pandit et al., 2010).

### Conflict of interest statement

The authors declare that the research was conducted in the absence of any commercial or financial relationships that could be construed as a potential conflict of interest.
